# *Mycobacterium tuberculosis* Transmission among Elderly Persons, Yamagata Prefecture, Japan, 2009–2015

**DOI:** 10.3201/eid2303.161571

**Published:** 2017-03

**Authors:** Junji Seto, Takayuki Wada, Yu Suzuki, Tatsuya Ikeda, Katsumi Mizuta, Taro Yamamoto, Tadayuki Ahiko

**Affiliations:** Yamagata Prefectural Institute of Public Health, Yamagata, Japan (J. Seto, Y. Suzuki, T. Ikeda, K. Mizuta, T. Ahiko);; Nagasaki University, Nagasaki, Japan (T. Wada, T. Yamamoto)

**Keywords:** contact investigation, contact tracing, elderly persons, Mycobacterium tuberculosis, tuberculosis and other mycobacteria, TB, bacteria, respiratory infections, variable-number tandem-repeat analysis, VNTR, Japan, transmission, epidemiology, molecular epidemiology, reactivation, latent infection

## Abstract

In many countries with low to moderate tuberculosis (TB) incidence, cases have shifted to elderly persons. It is unclear, however, whether these cases are associated with recent *Mycobacterium tuberculosis* transmission or represent reactivation of past disease. During 2009–2015, we performed a population-based TB investigation in Yamagata Prefecture, Japan, using in-depth contact tracing and 24-loci variable-number tandem-repeat typing optimized for Beijing family *M. tuberculosis* strains. We analyzed 494 strains, of which 387 (78.3%) were derived from elderly patients. Recent transmission with an epidemiologic link was confirmed in 22 clusters (70 cases). In 17 (77.3%) clusters, the source patient was elderly; 11 (64.7%) of the 17 clusters occurred in a hospital or nursing home. In this setting, the increase in TB cases was associated with *M. tuberculosis* transmissions from elderly persons. Prevention of transmission in places where elderly persons gather will be an effective strategy for decreasing TB incidence among predominantly elderly populations.

The World Health Organization End TB strategy (*1*) calls for every country, depending on their tuberculosis (TB) situation, to accelerate efforts designed to end TB. In Japan, 14.4 TB cases/100,000 population were reported in 2015. A small percentage of those cases occurred in foreign-born (6.4%) and HIV-positive (<0.1%) persons, who thus are not currently considered to pose a transmission threat; however, 71.8% of the reported cases were in elderly persons (>60 years of age) (*1*,*2*). Given that the incidence of TB was high in Japan until the 1970s (*3*), the current elderly population is regarded as vulnerable to TB onset from reactivation of remotely acquired latent infection (*4*,*5*). However, TB transmission among elderly populations has not been determined worldwide (*6*).

Molecular epidemiology can help identify recent TB transmission and, thereby, contribute to the creation of specific intervention programs for advanced TB control. Variable-number tandem-repeat (VNTR) typing is a useful method for rapidly detecting TB infections caused by the same strain of *Mycobacterium tuberculosis* (*7*); such cases might occur from recent transmission. A combination of VNTR typing and in-depth contact tracing has detected previously unrecognized recent TB transmission in various settings (*8*–*11*). However, data are scarce from areas such as Japan that might include large numbers of patients with reactivated TB (i.e., reactivation of past latent TB infection because of a weakened immune system).

The Beijing family, within lineage 2 (East Asian) of *M. tuberculosis*, has shown cumulative microevolution while spreading globally from its origin in eastern Asia (*12*–*16*). Worldwide, the modern Beijing subfamily predominates (*16*), but in Japan, the ancient Beijing subfamily, which is subdivided into 4 sublineages (ST11/26, STK, ST3, and ST25/19), accounts for >75% of Beijing family strains (*17*–*19*). Thus, appropriate VNTR subsets were proposed to discriminate Beijing *M. tuberculosis* lineages (*20*,*21*); a 24-loci subset, named 24_Beijing_, is used to achieve high discriminatory power for *M. tuberculosis* Beijing clinical isolates in Japan (*19*,*21*). The subset comprises 15-loci mycobacterial interspersed repetitive units (MIRUs) (*7*) and 9 additional loci, including 3 hypervariable loci (*21*).

We performed a population-based TB investigation in Yamagata Prefecture, Japan, combining 24_Beijing_-VNTR typing and in-depth contact tracing. We aimed to clarify the overall picture of recent *M. tuberculosis* transmission and thereby contribute to preventing the spread of TB in Yamagata Prefecture, where the annual TB incidence during the past decade was 7–13 cases/100,000 persons (*3*) and where 80.5% of reported patients were >60 years of age in 2015 (*2*). The investigation was designed to provide insight about TB among predominantly elderly populations as an aid to countries experiencing an increase in TB cases among the elderly (*1*,*22*,*23*). This work was approved by the Ethics Committees of Yamagata Prefectural Institute of Public Health (approval no. YPIPHEC H24–04 and YPIPHEC 16–08) and of the Institute of Tropical Medicine, Nagasaki University (approval no. 130606112).

## Materials and Methods

### Study Setting

Yamagata Prefecture, located in the northern part of Japan’s main island of Honshu, is subdivided into 4 topographically separated areas, each of which has a public health center (Technical Appendix 1 Figure 1). In 2014, Yamagata Prefecture had 1.1 million inhabitants, of whom 38.2% were elderly (>60 years of age) and 0.5% were noncitizen residents. Statistical data used in the study was provided by the Statistics Planning Division, Yamagata Prefecture.

### Study Population

The study included most patients in Yamagata Prefecture with culture-confirmed TB reported during January 1, 2009–December 31, 2015. On the basis of Japan’s Law Regarding Infectious Disease Prevention and Medical Care for the Patients, which was implemented on April 1, 1999, public health centers collect *M. tuberculosis* strains isolated from patients in order to conduct molecular investigations. During 2009–2015, public health centers routinely delivered collected strains to the Yamagata Prefectural Institute of Public Health within 2 months after notification of TB cases. These collected strains corresponded in part to those described in our earlier works, which mainly analyzed genetic features of *M. tuberculosis* (e.g., phylogenetic classification and the genome sequence) (*19*,*24*,*25*).

### Genotyping

We usually finished 24_Beijing_-VNTR typing within 3 days after arrival of the strains. We confirmed the amplified PCR fragment sizes by using a microchip electrophoresis system (MCE–202; Shimadzu Corp., Kyoto, Japan) and agarose gel electrophoresis. We calculated the number of repeats for each locus from the sizes of PCR products, in agreement with published allelic tables (*26*). For this study, we defined a preliminary TB cluster when the 24_Beijing_-VNTR profile of a strain was a single-locus variant (SLV) or was indistinguishable from that of other strains.

We estimated *M. tuberculosis* lineages of the clinical isolates by using maximum a posteriori estimation with the 24_Beijing_-VNTR profile, as described previously (*19*). We divided the strains into 6 lineages: the group of non-Beijing *M. tuberculosis* lineages, 4 sublineages (ST11/26, STK, ST3, and ST25/19) of ancient Beijing subfamily, and the modern Beijing subfamily.

### Data Collection

Public health centers routinely collect demographic (age, sex, country of birth, and address), clinical (site of disease, acid-fast bacilli sputum smear status, and treatment history), epidemiologic (family members, occupation, and contacts during onset), and microbiologic (culture and drug-susceptibility status of *M. tuberculosis* strain) characteristics for all reported TB patients. Public health centers use these data to determine whether interferon-γ release assays and chest radiography should be used to determine whether contacts of patients have latent TB infection. When a preliminary TB cluster is confirmed, public health nurses investigate the behavior history of patients within clusters to determine recent *M. tuberculosis* transmission. If patients in a cluster live in dispersed areas, the investigations are performed in cooperation with the responsible public health centers. In addition, after November 2013, public health centers asked all patients with culture-confirmed TB to complete a long or short version of a self-administered questionnaire that specifically elicits responses associated with residence, travel history, transportation, and places of social aggregation (Technical Appendix 2). Public health centers decide which version of the questionnaire to use, depending on patient willingness and ability to fill out the form. For example, in this study, the short version was used for elderly TB patients (especially those >80 years of age) who exhibited forgetfulness, tremulousness of hands, or an unwillingness to complete the questionnaire. Using social interaction data gathered from the case investigations and questionnaires, public health centers graded patients within clusters as epidemiologically linked (i.e., patients had shared space at the same time); possibly linked (i.e., patients had shared space but not at the same time); or not linked (i.e., no shared space was found for patients), according to the classification method of Walker et al. (*27*).

### Cluster Analysis

We defined cases as a cluster when their 24_Beijing_-VNTR profiles were indistinguishable from each other or when a social interaction was graded as epidemiologically linked or possibly linked in the SLV group. We excluded nonlinked cases in the SLV group from the cluster, based on the assertion by Allix-Béguec et al. that “even for hypervariable loci, at least in the absence of further specific epidemiological or contact tracing evidence, the definition of molecular clustering should remain restricted to full identity of the markers” (*28*). By comparing 24-loci MIRU-VNTR profiles proposed by Supply et al. (*7*) and results of whole-genome sequencing, Walker et al. (*27*) showed that *M. tuberculosis* strains in the SLV group contained many more single-nucleotide polymorphisms than those in the indistinguishable group.

We calculated the proportion of clustered cases resulting from recent *M. tuberculosis *transmission by using the *n* − 1 method, defined as (*N*_c _− *n*_c_)/*N*_o_, where *N*_c_ stands for the total number of clustered cases, *n*_c_ signifies the number of clusters (i.e., equal to the number of source cases), and *N*_o_ denotes the total number of cases in this study (*9*,*29*). In addition, we calculated the percentage of cases resulting from epidemiologic links, including only cases and clusters with confirmed links in the calculation [(no. of clustered cases with epidemiologic links − no. of clusters with epidemiologic links)/*N*_o_] (*9*). We visualized cluster diagrams displaying epidemiologic links by using Cytoscape 3.3.0, an open-source bioinformatics software platform (*30*).

### Statistical Analysis

We calculated odds ratios and 95% CIs by using logistic regression analysis. To determine the association between cluster formation and epidemiologic features (especially age groups), we applied multivariate logistic regression analysis using, for example, age group, sex, and *M. tuberculosis* lineage as explanatory variables. We used backward, stepwise variable selection to select the multivariate model with a probability entry of <0.2. Residual analysis was used when logistic regression analysis was not applicable. We considered p<0.05 as statistically significant. All statistical analyses were conducted using R version 3.0.2 (R Foundation for Statistical Computing, Vienna, Austria).

## Results

During 2009–2015, a total of 854 TB cases were reported in Yamagata Prefecture; 676 of the cases were diagnosed as pulmonary TB, of which 513 were culture-confirmed. We studied 494 (57.8%) of the 854 cases (469 [91.4%] of the 513 pulmonary cases and 25 extrapulmonary cases). We collected *M. tuberculosis* strains from the patients and determined the 24_Beijing_-VNTR profiles. Patients had a mean (±SD) age of 72.3 ± 19.9 (range 18–100) years. Most patients were >60 years of age (387 patients, 78.3%), Japanese (478 patients, 96.8%), and undergoing initial TB treatment (466 patients, 94.3%), and most had pulmonary symptoms (469 patients, 94.9%) and non–multidrug-resistant TB (491 patients, 99.4%). No patients were HIV-positive, illicit drug users, or homeless.

Results of 24_Beijing_-VNTR typing showed that 173 strains formed 52 preliminary clusters, which were aggregations of indistinguishable and SLV profiles (Technical Appendix 1 Table 1). Of note, the proportion of nonlinked cases in the SLV group was remarkably high (45/57, 78.9%) when we separated cases belonging to the preliminary cluster into indistinguishable and SLV groups (Table 1). Considering these findings and the assertion of Allix-Béguec et al. (*28*), we excluded 45 cases for which 24_Beijing_-VNTR profiles formed SLV clusters without epidemiologic links. The remaining 128 cases formed 42 clusters (Technical Appendix 1 Table 1), each of which contained 2–17 cases; 45.3% (58/128) of cases were in small clusters (2 cases), 25.0% (32/128) were in medium clusters (3 or 4 cases), and 29.7% (38/128) were in 3 large clusters (7, 14, and 17 cases, respectively). Cases attributable to recent transmission accounted for 17.4% (128 clustered cases − 42 clusters/494 total cases).

**Table 1:** Crude odds ratio for single-locus variant among 173 *Mycobacterium tuberculosis* strains forming preliminary clusters, by epidemiologic links of tuberculosis cases, Yamagata Prefecture, Japan, 2009–2015

Epidemiologic link	24_Beijing_-VNTR profile, no. (%)		Crude odds ratio (95% CI)*
	Indistinguishable, n = 116	Single-locus variant, n = 57	
Linked	42 (36.2)	11 (19.3)	1.0
Possibly linked	16 (13.8)	1 (1.8)	0.2 (0.03–2.0)
Not linked	58 (50.0)	45 (78.9)	**3.0 (1.4–6.4)**

*CIs that do not overlap the null value of odds ratio = 1 are shown in bold.

In both univariate and multivariate analyses, odds ratios for cluster formation were lower for patients >60 years of age than those <39 of age (Table 2). However, among the *M. tuberculosis* lineages, only ancient Beijing lineage ST11/26, which was represented by cluster 12, the largest cluster (n = 17; Technical Appendix 1 Table 1), showed a markedly high odds ratio against the modern Beijing subfamily in both univariate and multivariate analyses. The odds ratios of ancient Beijing lineages STK and ST3 against the modern Beijing lineage were significant only with univariate analysis. We also determined the risk for infection with the different *M. tuberculosis* lineages by age group (Table 3). The proportion of infections with STK, ST3, and ST25/19 was remarkably high among patients >60 years of age, whereas the proportion of infections with the modern Beijing subfamily was significantly higher (p<0.01) among patients <59 years of age.

**Table 2:** Odds ratio for cluster formation among 494 persons with tuberculosis, Yamagata Prefecture, Japan, 2009–2015*

Patient characteristic	24_Beijing_-VNTR profile, no. (%)			Odds ratio (95% CI)†	
	Not clustered, n = 366	Clustered, n = 128		Univariate	Multivariate‡
Age group					
<39	31 (8.5)	30 (23.4)		1.0	1.0
40–59	25 (6.8)	21 (16.4)		0.9 (0.4–1.9)	0.8 (0.3–1.8)
60–79	96 (26.2)	35 (27.3)		**0.4 (0.2–0.7)**	**0.4 (0.2–0.8)**
>80	214 (58.5)	42 (32.8)		**0.2 (0.1–0.4)**	**0.2 (0.1–0.4)**
Sex					
F	143 (39.1)	59 (46.1)		1.3 (0.9–2.0)	1.6 (0.99–2.4)
M	223 (60.9)	69 (53.9)		1.0	1.0
Birthplace					
Japan	351 (95.9)	127 (99.2)		5.4 (0.7–41.5)	**16.7 (2.0–137.0)**
Other	15 (4.1)	1 (0.8)		1.0	1.0
Site of disease					
Pulmonary, sputum smear–positive	251 (68.6)	78 (60.9)		1.0	–
Pulmonary, sputum smear–negative	97 (26.5)	44 (34.4)		1.5 (0.9–2.3)	–
Extrapulmonary	18 (4.9)	6 (4.7)		1.1 (0.4–2.8)	–
Treatment history					
Initial	343 (93.7)	123 (96.1)		1.6 (0.6–4.4)	–
Retreatment	23 (6.3)	5 (3.9)		1.0	–
*M. tuberculosis* lineage					
Non-Beijing	102 (27.9)	38 (29.7)		0.7 (0.4–1.4)	1.2 (0.6–2.3)
ST11/26	14 (3.8)	19 (14.8)		**2.7 (1.2–6.3)**	**2.5 (1.02–6.1)**
STK	72 (19.7)	10 (7.8)		**0.3 (0.1–0.6)**	0.4 (0.2–1.1)
ST3	68 (18.6)	15 (11.7)		**0.4 (0.2–0.9)**	0.8 (0.3–1.7)
ST25/19	60 (16.4)	21 (16.4)		0.7 (0.4–1.4)	1.0 (0.5–2.2)
Modern Beijing	50 (13.7)	25 (19.5)		1.0	1.0

*–, no variables. †CIs that do not overlap the null value of odds ratio = 1 are shown in bold. ‡Adjusted for the other factors used in the multivariate model.

**Table 3:** Lineages of 494 *Mycobacterium tuberculosis* strains by patient age group in Yamagata Prefecture, Japan, 2009–2015

Patient age group, y	No. (%) patients	*M. tuberculosis* lineage, no. (%)					
		Non-Beijing	Ancient Beijing				Modern Beijing
			ST11/26	STK	ST3	ST25/19	
<39	61 (100)	16 (26.2)	13 (21.3)*	4 (6.6)	2 (3.3)	3 (4.9)	23 (37.7)*
40–59	46 (100)	12 (26.1)	4 (8.7)	2 (4.3)	1 (2.2)	10 (21.7)	17 (37.0)*
60–79	131 (100)	37 (28.2)	6 (4.6)	19 (14.5)	24 (18.3)	30 (22.9)†	15 (11.5)
>80	256 (100)	75 (29.3)	10 (3.9)	57 (22.3)*	56 (21.9)*	38 (14.8)	20 (7.8)

*Significantly higher proportion by residual analysis (p<0.01). †Significantly higher proportion by residual analysis (p<0.05).

We confirmed epidemiologic links in 22 (52.4%) of the 42 TB clusters that occurred during 2009–2015; the linked cases consisted of 20 source cases (i.e., the source of *M. tuberculosis* transmission) and 50 secondary TB cases (Figure) (Technical Appendix 1 Figure 2). For each cluster, we identified a source case by information about the patients (e.g., the time of diagnosis, degree of infectiousness, the start of the infectious period based on sputum smear status, severity and duration of respiratory symptoms, findings on chest radiographs, and sociability of the patient). Source cases in clusters 12 and 34 were not included in this study because they occurred outside the study period or lacked a VNTR profile. The most common transmission setting was hospitals that had cared for TB patients (15 [30.0%] of 50 secondary cases), followed by households (14 [28.0%] of 50 secondary cases). We found unsuspected links for 23 (46.0%) of 50 secondary cases by conducting in-depth contact tracings after VNTR typing. Among the 23 cases, 22 (95.7%) aggregated with other TB cases in settings outside the household: hospitals (13 cases), pachinko parlors (7 cases), a nursing home (1 case), and a sporting event (1 case). The proportion of cases attributable to recent transmission after adjustment for epidemiologic links was 9.7% (70 clustered cases with epidemiologic links − 22 clusters with epidemiologic links/494 total cases). Furthermore, because unsuspected transmission settings were found within clusters 03, 12, and 34 after VNTR typing, we performed interferon-γ release assays on samples from close contacts of TB case-patients in those settings; none of the results were positive (data not shown).

**Figure:**
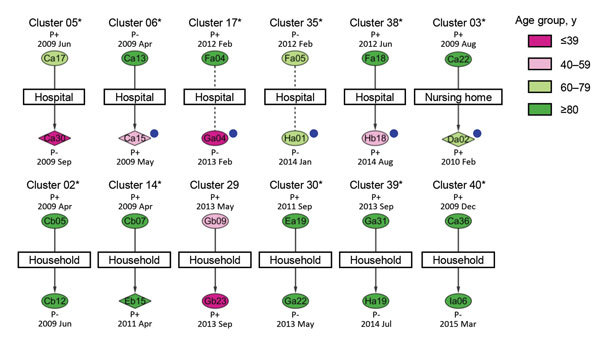
Twelve small tuberculosis (TB) clusters (2 cases each) among a total of 22 clusters with epidemiologic links between patients, Yamagata Prefecture, Japan, 2009–2015. Ovals and diamonds represent individual cases, by patient age group, in each cluster; numbers inside symbols are patient identification codes. Ovals indicate cases with an indistinguishable 24-loci variable-number tandem-repeat typing profile optimized for Beijing family *M. tuberculosis* strains (24_Beijing_-VNTR profile); diamonds indicate cases with a single-locus variant profile. Vertical arrows and dotted lines between cases within a cluster indicate linked and possibly linked cases, respectively. Transmission settings for linked cases are shown within rectangles. Case notification dates and patient disease sites are shown above/below the case symbol; P+ and P− indicate pulmonary smear–positive and ­–negative cases, respectively. Blue dots indicate confirmation of the epidemiologic link by in-depth contact tracings after 24_Beijing_-VNTR typing. Asterisks indicate clusters that began with a TB source patient who was >60 years of age. An expanded version of this figure is available as Technical Appendix 1 Figure 2).

For the large clusters (clusters 12, 26, and 34), we confirmed that there had been a delay between symptom onset and diagnosis for the source patients or that the source patients were highly socially active (Technical Appendix 1 Table 2). In cluster 12, we studied 17 cases reported during 2009–2015; another 18 cases were excluded from the study because they occurred outside the study period (Technical Appendix 1 Figure 2). In cluster 34, a diagnosis of TB in the probable source case-patient was missed because the patient had been diagnosed with lung cancer. However, a typical tuberculous cavity was found by retrospective viewing of a chest radiograph taken before the patient’s death.

Seventeen clusters originating from elderly patients were of small or medium size; the exception was cluster 34, for which the source case had not been diagnosed as TB (Technical Appendix 1 Figure 2). Source cases of the clusters aggregated with secondary cases in hospitals (9 clusters), households (7 clusters), and nursing homes (2 clusters). Almost all of the secondary case-patients aggregating at households and nursing homes were elderly persons, whereas most secondary case-patients within the hospital clusters were younger persons. Twelve (80.0%) of 15 secondary cases in hospital settings were in women <59 years of age, including 11 patients who were working as nurses or nurse’s aides.

To investigate risk factors for nonlinked results of contact tracings, we analyzed characteristics of 128 clustered cases (Table 4). Multivariate analysis showed that elderly persons and *M. tuberculosis* lineages (non-Beijing and ancient Beijing) were independent risk factors for nonlinked results of contact tracings.

**Table 4:** Risk factors for nonlinked results of contact tracings among 128 TB cases clustered in Yamagata Prefecture, Japan, 2009–2015

Characteristic	No. (%) cases with epidemiologic link			Odds ratio (95% CI)*	
	Linked or possibly linked	Not linked		Univariate	Multivariate†
Patient age group					
<59	37 (52.9)	14 (24.1)		1.0	1.0
>60	33 (47.1)	44 (75.9)		**3.5 (1.6–7.6)**	**2.9 (1.3–6.6)**
Patient sex					
F	39 (55.7)	20 (34.5)		1.0	1.0
M	31 (44.3)	38 (65.5)		**2.4 (1.2–4.9)**	2.2 (0.994–4.9)
*Mycobacterium tuberculosis* lineage					
Non-Beijing	16 (22.9)	22 (37.9)		**15.8 (3.3–76.9)**	**12.5 (2.5–63.3)**
Ancient Beijing	31 (44.3)	34 (58.6)		**12.6 (2.7–57.9)**	**10.1 (2.1–48.2)**
Modern Beijing	23 (32.9)	2 (3.4)		1.0	1.0

*CIs that do not overlap the null value of odds ratio = 1 are shown in bold. †Adjusted for the other factors used in the multivariate model.

## Discussion

We conducted molecular and epidemiologic TB investigations among a population in which 78.3% of the patients were >60 years of age. Among 22 TB clusters with epidemiologic links, 17 (77.3%) were traced to an elderly source patient (Technical Appendix 1 Figure 2). Of those 17 clusters, 11 occurred in hospitals and nursing homes. Transmission in these settings involved secondary cases mostly among younger persons. Our findings indicate that elderly persons must be included in TB prevention and control measures in countries with low to moderate incidence that have experienced a shifting of TB cases toward the elderly (*1*,*22*,*23*).

Our results suggest that elderly patients with TB are a source of TB spread involving younger persons. Borgdorff et al. showed that the number of secondary cases generated per source case decreased concomitantly with increasing age of the source patient; source cases involving elderly persons tend to form clusters of TB cases among elderly persons (*6*). Our findings show that 16 of 17 clusters that began with an elderly source patient were small or medium size; however, cluster 34, caused by an elderly person who was not diagnosed with TB, formed a large cluster. Eleven clusters that had an elderly source patient included transmissions in hospitals and nursing homes, and 80% of secondary cases in hospital settings were in persons <59 years of age. Elderly patients with TB often lack typical symptoms, such as cough and fever (*31*,*32*). In addition, diagnosis of TB in elderly persons might be delayed because they sometimes have multiple underlying diseases such as aspiration pneumonia and chronic obstructive pulmonary disease. Given that various factors may delay TB diagnosis in elderly persons, efforts should be taken to decrease such delays in order to stop or control the spread of TB. Furthermore, information regarding TB prevention should be provided to workers who have close and routine contact with elderly persons (e.g., hospital and nursing home staff).

Contact investigations are necessary to acquire the greatest public health benefit for most infectious diseases. Our findings confirmed unsuspected links in 46.0% of secondary TB cases through in-depth contact tracing after VNTR typing (Technical Appendix 1 Figure 2). In addition, we sought undiscovered latent TB infection cases by using interferon-γ release assays to find patient contacts in unsuspected settings. Given that earlier studies performed similar investigations and found latent and active TB cases (*8*,*9*,*11*), a combination of population-based molecular typing and further contact investigation is expected to be an effective strategy for discovering unknown latent TB infections or active TB cases. Moreover, we examined links in detail for 57 patients infected with *M. tuberculosis* strains in the SLV group; our findings confirmed epidemiologic links in 21.1% (12/57) of the cases (Table 1). These results indicate that in-depth contact tracings should be performed for TB cases caused by *M. tuberculosis* strains with SLV profiles.

The results of our study suggest that clusters that include elderly persons without epidemiologic links might be caused by the past endemic *M. tuberculosis* strains. We estimated, on the basis of molecular typing (*9*), that 17.4% (128 clustered cases − 42 clusters/494 total cases) of TB cases in this study were attributable to recent transmission; however, after we made adjustment for epidemiologic links, only 9.7% (70 clustered cases with epidemiologic links − 22 clusters with epidemiologic links/494 total cases) of the cases were attributable to recent transmission. The main cause for this difference in case numbers is that epidemiologic links were difficult to confirm for clustered cases among elderly persons (Table 4). In Japan, cases of TB among the elderly are attributable mainly to the endogenous relapse of *M. tuberculosis* infections that occurred near the time of World War II, when TB was highly prevalent (*4*,*5*). This fact suggests that past endemic strains, having indistinguishable VNTR profiles, have been isolated from elderly persons who have onset of TB in modern times caused by reactivation of latent TB infection. The confirmation of settings with recent *M. tuberculosis* transmission can guide interventions to control the spread of TB; however, the lack of such confirmation, despite in-depth contact tracings, may suggest infection with the past endemic *M. tuberculosis* strain, and the transmission settings for such infections can be difficult to detect.

Epidemiologic data together with phylogenetic information for *M. tuberculosis* strains causing infections might be useful in determining the time of TB infection. In this study, more than half of the strains were of the ancient Beijing subfamily, and the proportion of several sublineages from this family were markedly high in persons >60 years of age, whereas modern Beijing subfamily strains were prevalent in younger age groups (Table 3). Given that an earlier Japanese study found a similar tendency (*17*), predominant lineages will have shifted over time, at least in Japan. Such historical dynamics might provide valuable clues for estimating the background of isolated strains. For example, if nonclustered strains with STK or ST3 were isolated from elderly patients in Japan, then it might be reasonable that such cases were regarded as sporadic because of reactivation.

Our study had several potential limitations. First, contact tracings might not clarify all epidemiologic links among clustered TB cases. In particular, findings of the true location of transmission for young and active persons with TB may be missed because those persons tended to frequent many locations. By contrast, elderly persons with TB frequented fewer locations, but their disabilities (e.g., forgetfulness and impaired hearing) or death immediately after TB diagnosis compromised our data gathering. To overcome this limitation, the use of whole-genome sequencing, which has higher discriminatory power than VNTR typing, might indicate whether clustered strains are derived from recent transmission (*27*,*33*). Second, *M. tuberculosis* genotyping studies cannot investigate culture-negative TB cases. This unavoidable limitation caused a decrease of overall coverage in our study (i.e., >40% of TB cases were beyond the scope of investigation). However, our comprehensive investigation of culture-confirmed TB cases, which are more infectious than culture-negative cases (*34*) and form the basis of TB transmission, may have been sufficient for determining the representative transmission settings in our study area. Our findings from Yamagata Prefecture provide empiric evidence that nonhousehold settings populated or frequented by elderly persons (e.g., hospitals and social gathering settings) are hotspots for *M. tuberculosis* transmission among this population. The last limitation is that our study was restricted to a local setting. However, Theron et al. (*35*) recently proposed that an important factor for ending TB epidemics is to emphasize strategies at the local level, where TB transmission occurs. The accumulation of empiric evidence for various other local settings in Japan is expected to become a higher priority for decision-making for nationwide policies regarding TB.

In summary, molecular genotyping methods make it possible to perform evidence-based TB control (*8*–*11*,*27*). We confirmed the effectiveness of these methods in a mostly elderly population by using VNTR typing with in-depth contact tracing. Our results suggest that prevention of *M. tuberculosis* transmissions in places where elderly persons gather can be an effective strategy for decreasing TB incidence. A combination of molecular and epidemiologic data can assist public health officials in obtaining an overview of recent transmission and in detecting unsuspected transmission settings, thereby enabling further informational activities and interventions to prevent the spread of TB.

Technical Appendix 1 Details and characteristics of tuberculosis clusters formed by 24-loci variable-number tandem-repeat typing optimized for Beijing family *Mycobacterium tuberculosis* strains, Yamagata Prefecture, Japan, 2009–2015, and geographic location of Yamagata Prefecture.

Technical Appendix 2 Self-administered questionnaires used to elicit responses related to residential and travel history, transportation, and places of social aggregation from persons with culture-confirmed tuberculosis, Yamagata Prefecture, Japan, 2009–2015.
