# Supplementary material for: Mycobacterium tuberculosis Transmission among Elderly Persons, Yamagata Prefecture, Japan, 2009–2015
Source: Emerg Infect Dis. 2017 Mar;23(3):448–55. doi: 10.3201/eid2303.161571 (PMC5382749; doi:10.3201/eid2303.161571)
Supplement: Technical Appendix 1 — Details and characteristics of tuberculosis clusters formed by 24-loci variable-number tandem-repeat typing optimized for Beijing family Mycobacterium tuberculosis strains, Yamagata Prefecture, Japan, 2009–2015, and geographic location of Yamagata Prefecture. [file 16-1571-Techapp-s1.pdf]

Article DOI: http://dx.doi.org/10.3201/eid2303.161571

Mycobacterium tuberculosis Transmission among Elderly Persons, Yamagata Prefecture, Japan, 2009–2015

Technical Appendix 1

Technical Appendix 1 **Table 1.** Genotyping results of 173 *Mycobacterium tuberculosis* strains forming preliminary clusters and epidemiologic data of clustered TB cases in Yamagata Prefecture, Japan, 2009–2015*

Preliminary cluster, strain no.†	Cluster	Isolate	Age, y	Sex	Residential area	Registration month/year	Epidemiologic link: Linked	Epidemiologic link: Possibly linked	Epidemiologic link: Not linked	VNTR profile of $24_{Beijing}$-VNTR‡	SLV	*M. tuberculosis* lineage
01												
1	–	Fa02	92	F	A	02/2012			x	3 4 2 3 3 4 3 5 4 4 7 5 3 2 5 4 3 5 7 7 8 12 12 11	x	ST25/19
2	–	Gb03	81	F	B	01/2013			x	3 4 2 3 3 4 3 7 4 4 7 5 3 2 5 4 3 5 7 6 8 12 12 11	x	ST25/19
3	01	Ha36	70	M	A	10/2014			x	3 4 2 3 3 4 3 7 4 4 7 5 3 2 5 4 3 5 7 7 8 12 12 11		ST25/19
4	01	Ha37	80	M	A	10/2014			x	3 4 2 3 3 4 3 7 4 4 7 5 3 2 5 4 3 5 7 7 8 12 12 11		ST25/19
02												
5	02	Cb05	82	F	B	04/2009	x			4 4 2 3 3 3 3 3 4 2 7 3 3 9 4 3 4 3 5 13 8 10 13 12		STK
6	02	Cb12	86	M	B	06/2009	x			4 4 2 3 3 3 3 3 4 2 7 3 3 9 4 3 4 3 5 13 8 10 13 12		STK
03												
7	03	Ca22	82	F	A	08/2009	x			4 7 2 3 3 3 3 3 4 2 7 5 4 8 4 3 3 4 7 7 8 3/13 16 6	x	STK
8	03	Da02	76	F	A	02/2010	x			4 7 2 3 3 3 3 3 4 2 7 5 4 8 4 3 3 4 7 7 8 13 16 6	x	STK
04												
9	04	Ca02	72	M	A	01/2009			x	4 4 2 3 3 3 4 4 4 4 7 5 3 8 3 3 3 4 7 8 5 14 14 11		Modern Beijing
10	04	Ca31	81	M	A	10/2009			x	4 4 2 3 3 3 4 4 4 4 7 5 3 8 3 3 3 4 7 8 5 14 14 11		Modern Beijing
11	04	Da17	83	F	A	08/2010		x		4 4 2 3 3 3 4 4 4 4 7 5 3 8 3 3 3 4 7 8 5 14 14 11		Modern Beijing
12	04	Ea28	50	F	A	11/2011		x		4 4 2 3 3 3 4 4 4 4 7 5 3 8 3 3 3 4 7 8 5 14 14 11		Modern Beijing
05												
13	05	Ca17	78	M	A	06/2009	x			3 4 2 4 3 4 3 6 4 4 7 5 3 2 5 4 3 5 8 10 8 16 12 8	x	ST25/19
14	05	Ca30	25	F	A	09/2009	x			3 4 2 4 3 4 3 6 4 4 7 5 3 2 5 4 3 5 8 10 13 16 12 8	x	ST25/19
06												

Preliminary cluster, strain no.†	Cluster	Isolate	Age, y	Sex	Residential area	Registration month/year	Epidemiologic link: Linked	Epidemiologic link: Possibly linked	Epidemiologic link: Not linked	VNTR profile of 24$_{Beijing}$-VNTR‡	SLV	*M. tuberculosis* lineage
15	06	Ca13	84	M	A	04/2009	x			4 4 2 3 3 3 3 7 4 4 7 5 3 10 5 2 3 4 7 10 5 15 12 8	x	ST25/19
16	06	Ca15	41	F	A	05/2009	x			4 4 2 3 3 3 3 7 4 4 7 5 3 10 5 2 3 4 7 10 5 15 13 8	x	ST25/19
17	–	Ha22	82	M	A	09/2014			x	4 4 2 3 3 3 3 7 4 4 7 5 3 10 5 2 3 4 7 10 5 15 12 9	x	ST25/19
07												
18	07	Ca38	50	M	A	12/2009			x	4 4 2 3 3 3 3 7 2 4 7 5 3 10 5 2 3 4 7 10 >20 15 12 8		ST25/19
19	07	Da04	80	M	A	03/2010			x	4 4 2 3 3 3 3 7 2 4 7 5 3 10 5 2 3 4 7 10 >20 15 12 8		ST25/19
08												
20	08	Ca28	63	M	A	09/2009			x	2 3 2 1 3 3 1 3 3 2 5 3 8 3 3 3 2 4 12 5 2 5 5 2		Non-Beijing
21	08	Dc05	93	F	C	05/2010			x	2 3 2 1 3 3 1 3 3 2 5 3 8 3 3 3 2 4 12 5 2 5 5 2		Non-Beijing
09												
22	–	Dc08	77	M	C	08/2010			x	4 4 2 3 3 3 3 7 4 4 7 4 3 8 5 3 3 4 7 10 8 14 12 5	x	ST25/19
23	09	Da03	77	M	A	02/2010			x	4 4 2 3 3 3 3 7 4 4 7 5 3 8 5 3 3 4 7 10 8 14 12 5		ST25/19
24	–	Da34	85	M	A	01/2010			x	4 4 2 3 3 3 3 7 4 4 7 5 3 8 5 3 3 4 7 10 8 15 12 5	x	ST25/19
25	09	Ha11	77	M	A	05/2014			x	4 4 2 3 3 3 3 7 4 4 7 5 3 8 5 3 3 4 7 10 8 14 12 5		ST25/19
10												
26	10	Ca03	84	F	A	01/2009		x		4 4 2 3 3 3 4 5 3 4 7 5 3 7 3 2 3 4 7 8 5 14 20 8		Modern Beijing
27	10	Ea04	45	F	A	01/2011		x		4 4 2 3 3 3 4 5 3 4 7 5 3 7 3 2 3 4 7 8 5 14 20 8		Modern Beijing
28	10	Ea13	42	M	A	04/2011	x			4 4 2 3 3 3 4 5 3 4 7 5 3 7 3 2 3 4 7 8 5 14 20 8		Modern Beijing
29	10	Ea17	49	F	A	08/2011	x			4 4 2 3 3 3 4 5 3 4 7 5 3 7 3 2 3 4 7 8 5 14 20 8		Modern Beijing
11												
30	–	Ca19	79	M	A	08/2009			x	2 3 2 1 3 3 1 3 3 2 5 4 5 4 3 3 2 4 12 5 2 5 5 2	x	Non-Beijing
31	11	Ca34	82	M	A	01/2009			x	2 3 2 1 3 3 1 4 3 2 5 4 5 4 3 3 2 4 12 5 2 5 5 2		Non-Beijing
32	11	Ea16	83	M	A	07/2011			x	2 3 2 1 3 3 1 4 3 2 5 4 5 4 3 3 2 4 12 5 2 5 5 2		Non-Beijing
33	11	Ia15	79	M	A	08/2015			x	2 3 2 1 3 3 1 4 3 2 5 4 5 4 3 3 2 4 12 5 2 5 5 2		Non-Beijing
12												
34	12	Ca14	72	M	A	05/2009			x	3 4 2 2 3 3 4 5 4 4 7 4 3 8 4 3 3 2 14 10 8 12 9 7		ST11/26
35	12	Ca18	29	M	A	07/2009		x		3 4 2 2 3 3 4 5 4 4 7 4 3 8 4 3 3 2 14 10 8 12 9 7		ST11/26
36	12	Ca21	88	F	A	08/2009	x			3 4 2 2 3 3 4 5 4 4 7 4 3 8 4 3 3 2 14 10 8 12 9 7		ST11/26
37	12	Ca23	43	M	A	08/2009		x		3 4 2 2 3 3 4 5 4 4 7 4 3 8 4 3 3 2 14 10 8 12 9 7		ST11/26
38	12	Ca25	35	M	A	02/2009	x			3 4 2 2 3 3 4 5 4 4 7 4 3 8 4 3 3 2 14 10 8 12 9 8	x	ST11/26
39	12	Ca32	31	M	A	10/2009		x		3 4 2 2 3 3 4 5 4 4 7 4 3 8 4 3 3 2 14 10 8 12 9 7		ST11/26
40	12	Da07	26	F	A	03/2010		x		3 4 2 2 3 3 4 5 4 4 7 4 3 8 4 3 3 2 14 9 8 12 9 7	x	ST11/26
41	12	Da14	24	M	A	07/2010			x	3 4 2 2 3 3 4 5 4 4 7 4 3 8 4 3 3 2 14 10 8 12 9 7		ST11/26
42	12	Ea03	72	F	A	01/2011			x	3 4 2 2 3 3 4 5 4 4 7 4 3 8 4 3 3 2 14 10 8 12 9 7		ST11/26
43	12	Ea18	27	M	A	09/2011		x		3 4 2 2 3 3 4 5 4 4 7 4 3 8 4 3 3 2 14 10 8 12 9 7		ST11/26
44	12	Ea31	36	M	A	12/2011	x			3 4 2 2 3 3 4 5 4 4 7 4 3 8 4 3 3 2 14 10 8 12 9 7		ST11/26

Preliminary cluster, strain no.†	Cluster	Isolate	Age, y	Sex	Residential area	Registration month/year	Epidemiologic link: Linked	Epidemiologic link: Possibly linked	Epidemiologic link: Not linked	VNTR profile of $24_{Beijing}$-VNTR‡	SLV	*M. tuberculosis* lineage
45	12	Ea33	30	F	A	07/2011			x	3 4 2 2 3 3 4 5 4 4 7 4 3 8 4 3 3 2 14 10 8 12 9 7		ST11/26
46	12	Fa25	36	M	A	07/2012			x	3 4 2 2 3 3 4 5 4 4 7 4 3 8 4 3 3 2 14 10 8 12 9 7		ST11/26
47	12	Ga06	32	M	A	03/2013			x	3 4 2 2 3 3 4 5 4 4 7 4 3 8 4 3 3 2 14 10 8 12 9 7		ST11/26
48	12	Ga08	57	F	A	03/2013	x			3 4 2 2 3 3 4 5 4 4 7 4 3 8 4 3 3 2 14 10 8 12 9 7		ST11/26
49	12	Ga18	77	M	A	04/2013			x	3 4 2 2 3 3 4 5 4 4 7 4 3 8 4 3 3 2 14 10 8 12 9 7		ST11/26
50	12	Ib16	39	M	B	09/2015		x		3 4 2 2 3 3 4 5 4 4 7 4 3 8 4 3 3 2 14 10 8 12 9 7		ST11/26
51	–	Ia21	53	F	A	01/2015			x	3 4 2 2 3 3 4 5 4 4 7 4 3 9 4 3 3 2 14 10 8 12 9 7	x	ST11/26
13												
52	13	Eb02	32	F	B	01/2011			x	3 4 2 3 3 4 3 7 4 4 7 5 3 2 5 4 3 5 7 10 8 10 12 9		ST25/19
53	–	Ea08	79	F	A	02/2011			x	3 4 2 3 3 4 3 7 4 4 6 5 3 2 5 4 3 5 7 10 8 10 12 12	x	ST25/19
54	13	Ea32	86	F	A	12/2011			x	3 4 2 3 3 4 3 7 4 4 7 5 3 2 5 4 3 5 7 10 8 10 12 9		ST25/19
55	13	Fb11	59	M	B	12/2012			x	3 4 2 3 3 4 3 7 4 4 7 5 3 2 5 4 3 5 7 10 8 10 12 9		ST25/19
56	–	Fa33	83	M	A	11/2012			x	3 4 2 3 3 4 3 7 4 4 7 5 3 2 5 4 3 5 7 10 8 12 12 12	x	ST25/19
57	–	Hb05	54	M	B	03/2014			x	3 4 2 3 3 4 3 7 4 4 7 5 3 2 5 4 3 5 7 13 8 10 12 12	x	ST25/19
58	–	Ha31	87	F	A	12/2014			x	3 4 2 3 3 4 3 7 4 4 7 5 3 2 5 4 3 5 7 10 8 10 12 12	x	ST25/19
14												
59	14	Cb07	83	M	B	04/2009	x			4 4 2 3 1 3 3 6 4 4 5 5 4 8 5 2 4 4 7 10 9 24 14 12	x	ST3
60	14	Eb15	85	F	B	04/2011	x			4 4 2 3 1 3 3 6 4 4 5 5 4 8 5 2 4 4 7 10 9 22 14 12	x	ST3
15												
61	15	Fa01	43	F	A	01/2012			x	2 3 2 1 3 3 1 4 3 2 5 3 5 3 3 3 2 4 12 5 2 5 9 2		Non-Beijing
62	15	Ga33	84	F	A	08/2013			x	2 3 2 1 3 3 1 4 3 2 5 3 5 3 3 3 2 4 12 5 2 5 9 2		Non-Beijing
16												
63	16	Db02	86	F	B	02/2010			x	4 4 2 3 1 3 3 7 4 4 7 5 3 8 5 2 4 4 7 10 9 16 14 13		ST3
64	16	Fa03	68	F	A	01/2012			x	4 4 2 3 1 3 3 7 4 4 7 5 3 8 5 2 4 4 7 10 9 16 14 13		ST3
17												
65	17	Fa04	82	M	A	02/2012		x		4 4 2 3 3 3 4 6 4 4 7 4 3 8 3 3 1 4 7 8 8 13 13 4		Modern Beijing
66	17	Ga04	37	F	A	02/2013		x		4 4 2 3 3 3 4 6 4 4 7 4 3 8 3 3 1 4 7 8 8 13 13 4		Modern Beijing
18												
67	18	Dc13	83	M	C	12/2010			x	2 4 2 1 3 3 1 4 3 2 5 3 5 5 3 3 2 4 12 5 2 4 5 2		Non-Beijing
68	–	Fa07	80	M	A	04/2012			x	2 4 2 1 3 3 1 4 3 2 5 3 5 5 3 3 2 4 12 5 2 3 5 2	x	Non-Beijing
69	18	Fa26	72	F	A	07/2012			x	2 4 2 1 3 3 1 4 3 2 5 3 5 5 3 3 2 4 12 5 2 4 5 2		Non-Beijing
19												
70	–	Da18	93	F	A	09/2010			x	2 4 2 1 2 3 1 4 3 2 5 3 7 4 3 3 2 4 10 5 2 5 5 2	x	Non-Beijing
71	–	Fa09	81	F	A	05/2012			x	2 4 2 1 2 3 1 4 3 2 5 3 5 4 3 3 2 4 10 5 2 5 5 2	x	Non-Beijing
72	–	Ha26	93	F	A	11/2014			x	2 4 2 1 2 3 1 4 3 2 4 3 5 4 3 3 2 4 10 5 2 5 5 2	x	Non-Beijing

Preliminary cluster, strain no.†	Cluster	Isolate	Age, y	Sex	Residential area	Registration month/year	Epidemiologic link: Linked	Epidemiologic link: Possibly linked	Epidemiologic link: Not linked	VNTR profile of $24_{Beijing}$-VNTR‡	SLV	*M. tuberculosis* lineage
20												
73	–	Da24	61	F	A	01/2010			x	4 4 2 3 1 3 3 7 4 4 12 4 3 8 5 2 4 4 7 9 9 13 14 9	x	ST3
74	–	Da27	78	F	A	11/2010			x	4 4 2 3 1 3 3 7 4 4 8 4 3 8 5 2 4 4 7 9 9 13 14 9	x	ST3
75	–	Fa16	85	M	A	04/2012			x	4 4 2 3 1 3 3 7 4 4 11 4 3 8 5 2 4 4 7 9 9 13 14 9	x	ST3
21												
76	19	Db05	79	M	B	04/2010			x	4 4 2 3 1 3 3 6 4 4 7 5 3 8 5 2 4 4 7 10 9 16 14 12		ST3
77	–	Eb07	86	M	B	07/2011			x	4 4 2 3 1 3 3 6 4 4 7 5 3 8 5 2 2 4 7 10 9 16 14 12	x	ST3
78	19	Ea21	85	M	A	01/2011			x	4 4 2 3 1 3 3 6 4 4 7 5 3 8 5 2 4 4 7 10 9 16 14 12		ST3
22												
79	–	Cb04	81	F	B	03/2009			x	3 4 2 3 3 4 3 7 4 4 7 5 3 2 5 4 3 5 7 10 13 9 12 11	x	ST25/19
80	–	Da09	82	M	A	04/2010			x	3 4 2 3 3 4 3 6 4 4 6 5 3 2 5 4 3 5 7 10 8 10 12 11	x	ST25/19
81	20	Da28	93	F	A	11/2010			x	3 4 2 3 3 4 3 7 4 4 7 5 3 2 5 4 3 5 7 10 8 9 12 11		ST25/19
82	20	Ga11	67	M	A	01/2013			x	3 4 2 3 3 4 3 7 4 4 7 5 3 2 5 4 3 5 7 10 8 9 12 11		ST25/19
83	–	Gb18	41	M	B	07/2013			x	3 4 2 3 3 4 3 7 4 4 7 5 3 2 5 4 3 5 7 10 8 13 12 11	x	ST25/19
84	20	Gc16	78	M	C	12/2013			x	3 4 2 3 3 4 3 7 4 4 7 5 3 2 5 4 3 5 7 10 8 9 12 11		ST25/19
85	–	Ha13	88	F	A	06/2014			x	3 4 2 3 3 4 3 6 4 4 7 5 3 2 5 4 3 5 7 10 8 10 12 11	x	ST25/19
86	–	Ia04	89	M	A	01/2015			x	3 4 2 3 3 4 3 7 4 4 7 5 3 2 5 4 3 5 7 10 8 14 12 11	x	ST25/19
87	–	Ib10	77	M	B	06/2015			x	3 4 2 3 3 4 3 6 4 4 7 5 3 2 5 4 3 5 7 10 8 9 12 11	x	ST25/19
23												
88	21	Ca37	32	F	A	12/2009	x			4 4 2 3 3 3 4 5 4 4 8 5 3 10 3 3 3 4 4 8 5 14 14 9		Modern Beijing
89	21	Fa21	39	F	A	09/2012	x			4 4 2 3 3 3 4 5 4 4 8 5 3 10 3 3 3 4 4 8 5 14 14 9		Modern Beijing
90	21	Fa38	44	M	A	12/2012	x			4 4 2 3 3 3 4 5 4 4 8 5 3 10 3 3 3 4 4 8 5 14 14 9		Modern Beijing
24												
91	–	Ea01	90	M	A	02/2011			x	2 4 2 4 2 3 2 3 3 2 5 3 3 8 3 4 2 4 7 5 >20 13 5 4	x	Non-Beijing
92	–	Fa22	80	F	A	06/2012			x	2 4 2 3 2 3 2 3 3/4 2 5 3 3 8 3 4 2 4 7 5 >20 13 5 4	x	Non-Beijing
25												
93	22	Fa23	38	F	A	06/2012			x	4 4 4 3 3 3 3 3 4 2 7 5 3 3 4 3 3 4 7 8 8 12 17 18		STK
94	22	Fa27	85	F	A	07/2012			x	4 4 4 3 3 3 3 3 4 2 7 5 3 3 4 3 3 4 7 8 8 12 17 18		STK
26												
95	–	Ca33	78	M	A	01/2009			x	4 4 2 3 3 3 3 6 4 4 7 5 3 10 5 2 3 4 7 10 >20 >20 12 8	x	ST25/19
96	–	Fa24	79	F	A	07/2012			x	4 4 2 3 3 3 3 6 4 4 7 5 3 10 5 2 3 4 7 10 >20 20 12 8	x	ST25/19
27												
97	23	Eb04	79	M	B	05/2011			x	3 4 1 2 5 1 2 2 3 4 1 3 3 8 4 1 5 2 15 6 >20 5 7 4		Non-Beijing
98	23	Fb04	72	F	B	01/2012			x	3 4 1 2 5 1 2 2 3 4 1 3 3 8 4 1 5 2 15 6 >20 5 7 4		Non-Beijing
28												

Preliminary cluster, strain no.†	Cluster	Isolate	Age, y	Sex	Residential area	Registration month/year	Epidemiologic link			VNTR profile of $24_{Beijing}$-VNTR‡	SLV	*M. tuberculosis* lineage
							Linked	Possibly linked	Not linked			
99	24	Fb07	43	F	B	07/2012			x	1 4 2 3 3 3 3 7 4 4 7 5 3 8 5 3 3 4 7 10 8 8 10 5		ST25/19
100	24	Fb08	76	M	B	09/2012			x	1 4 2 3 3 3 3 7 4 4 7 5 3 8 5 3 3 4 7 10 8 8 10 5		ST25/19
29												
101	25	Fb06	80	F	B	01/2012			x	4 4 4 2 2 2 0 7 3 2 5 3 1 6 3 3 2 4 16 0 5 7 3 4		Non-Beijing
102	25	Gb01	79	M	B	02/2013			x	4 4 4 2 2 2 0 7 3 2 5 3 1 6 3 3 2 4 16 0 5 7 3 4		Non-Beijing
30												
103	26	Fb05	26	M	B	11/2012	x			3 4 1 3 5 1 2 2 3 4 1 3 3 9 4 1 3 2 12 7 >20 6 9 4		Non-Beijing
104	26	Gb02	65	M	B	02/2013		x		3 4 1 3 5 1 2 2 3 4 1 3 3 9 4 1 3 2 12 7 >20 6 9 4		Non-Beijing
105	26	Gb04	49	F	B	01/2013	x			3 4 1 3 5 1 2 2 3 4 1 3 3 9 4 1 3 2 12 7 >20 6 9 4		Non-Beijing
106	26	Gb07	78	M	B	03/2013			x	3 4 1 3 5 1 2 2 3 4 1 3 3 9 4 1 3 2 12 7 >20 6 9 4		Non-Beijing
107	26	Gb08	70	M	B	04/2013			x	3 4 1 3 5 1 2 2 3 4 1 3 3 9 4 1 3 2 12 7 >20 6 9 4		Non-Beijing
108	26	Gb11	66	M	B	07/2013		x		3 4 1 3 5 1 2 2 3 4 1 3 3 9 4 1 3 2 12 7 >20 6 9 4		Non-Beijing
109	26	Gb17	36	F	B	07/2013	x			3 4 1 3 5 1 2 2 3 4 1 3 3 9 4 1 3 2 12 7 >20 6 9 4		Non-Beijing
110	26	Gb24	42	F	B	08/2013	x			3 4 1 3 5 1 2 2 3 4 1 3 3 9 4 1 3 2 12 7 >20 6 9 4		Non-Beijing
111	26	Gb26	30	M	B	09/2013			x	3 4 1 3 5 1 2 2 3 4 1 3 3 9 4 1 3 2 12 7 >20 6 9 4		Non-Beijing
112	26	Hb07	27	M	B	04/2014	x			3 4 1 3 5 1 2 2 3 4 1 3 3 6/9 4 1 3 2 12 7 >20 6 9 4	x	Non-Beijing
113	26	Hb21	51	F	B	11/2014			x	3 4 1 3 5 1 2 2 3 4 1 3 3 9 4 1 3 2 12 7 >20 6 9 4		Non-Beijing
114	26	Ib06	23	M	B	03/2015			x	3 4 1 3 5 1 2 2 3 4 1 3 3 9 4 1 3 2 12 7 >20 6 9 4		Non-Beijing
115	26	Ib11	19	M	B	07/2015	x			3 4 1 3 5 1 2 2 3 4 1 3 3 9 4 1 3 2 12 7 >20 6 9 4		Non-Beijing
116	26	Ib14	57	F	B	07/2015	x			3 4 1 3 5 1 2 2 3 4 1 3 3 9 4 1 3 2 12 7 >20 6 9 4		Non-Beijing
31												
117	–	Db16	64	M	B	11/2010			x	2 4 1 2 3 1 2 2 3 4 1 3 3 7 4 1 5 2 10 6 >20 5 6 5	x	Non-Beijing
118	27	Fb13	79	M	B	12/2012			x	2 4 1 2 3 1 2 2 3 4 1 3 3 7 4 1 5 2 13 6 >20 5 6 5		Non-Beijing
119	27	Ib07	78	M	B	03/2015			x	2 4 1 2 3 1 2 2 3 4 1 3 3 7 4 1 5 2 13 6 >20 5 6 5		Non-Beijing
32												
120	28	Eb01	86	M	B	01/2011			x	4 4 2 3 1 3 3 6 4 4 6 5 3 8 5 2 4 4 4 10 7 16 14 13		ST3
121	28	Ga14	80	F	A	03/2013			x	4 4 2 3 1 3 3 6 4 4 6 5 3 8 5 2 4 4 4 10 7 16 14 13		ST3
122	–	Ha21	91	F	A	08/2014			x	4 4 2 3 1 3 3 6 4 4 6 5 3 8 5 2 4 4 4 10 7 16 14 9	x	ST3
33												
123	–	Gc04	22	M	C	04/2013			x	4 4 2 3 3 3 4 6 4 4 7 5 3 8 3 3 3 4 7 8 8 14 12 10	x	Modern Beijing
124	29	Gb09	44	M	B	05/2013	x			4 4 2 3 3 3 4 6 4 4 7 5 3 8 3 3 3 4 7 8 8 14 15 10		Modern Beijing
125	29	Gb23	18	M	B	09/2013	x			4 4 2 3 3 3 4 6 4 4 7 5 3 8 3 3 3 4 7 8 8 14 15 10		Modern Beijing
34												
126	30	Ea19	83	M	A	09/2011	x			2 4 1 2 5 1 2 2 3 4 1 3 2 8 4 1 3 2 16 6 7 5 8 4		Non-Beijing
127	30	Ga22	80	F	A	05/2013	x			2 4 1 2 5 1 2 2 3 4 1 3 2 8 4 1 3 2 16 6 7 5 8 4		Non-Beijing

Preliminary cluster, strain no.†	Cluster	Isolate	Age, y	Sex	Residential area	Registration month/year	Epidemiologic link: Linked	Epidemiologic link: Possibly linked	Epidemiologic link: Not linked	VNTR profile of $24_{Beijing}$-VNTR‡	SLV	*M. tuberculosis* lineage
35												
128	–	Ca07	86	M	A	02/2009			x	2 4 2 1 3 3 1 2 3 2 5 3 5 5 3 3 2 4 13 5 2 5 5 2	x	Non-Beijing
129	–	Gb13	78	M	B	03/2013			x	2 4 2 1 3 3 1 2 3 2 5 3 5 5 3 3 2 4 10 5 2 5 5 2	x	Non-Beijing
36												
130	31	Gb10	80	M	B	06/2013	x			3 4 1 3 3 4 3 7 4 4 6 5 3 2 5 2 3 5 7 10 8 13 12 14/15	x	ST25/19
131	31	Gb16	79	M	B	08/2013	x			3 4 1 3 3 4 3 7 4 4 6 5 3 2 5 2 3 5 7 10 8 13 12 15		ST25/19
132	31	Hb13	80	F	B	07/2014	x			3 4 1 3 3 4 3 7 4 4 6 5 3 2 5 2 3 5 7 10 8 13 12 15		ST25/19
37												
133	32	Cc03	74	M	C	02/2009			x	4 4 2 3 1 3 3 7 4 4 7 5 3 8 5 2 4 4 7 10 9 14 14 21		ST3
134	32	Ga28	74	F	A	08/2013	x			4 4 2 3 1 3 3 7 4 4 7 5 3 8 5 2 4 4 7 10 9 14 14 21		ST3
135	32	Ga39	82	F	A	10/2013	x			4 4 2 3 1 3 3 7 4 4 7 5 3 8 5 2 4 4 7 10 9 14 14 21		ST3
38												
136	–	Eb09	84	M	B	09/2011			x	5 4 2 3 1 3 3 7 4 4 7 4 3 8 5 2 4 4 7 9 9 16 16 9	x	ST3
137	–	Gc10	66	M	C	08/2013			x	4 4 2 3 1 3 3 7 4 4 7 4 3 8 5 2 4 4 7 9 9 16 16 9	x	ST3
39												
138	33	Fb03	37	M	B	07/2012	x			2 4 2 3 2 3 2 3 3 2 5 3 3 6 3 4 2 4 3 5 >20 14 5 3		Non-Beijing
139	33	Gb21	68	F	B	09/2013	x			2 4 2 3 2 3 2 3 3 2 5 3 3 6 3 4 2 4 3 5 >20 14 5 3		Non-Beijing
140	33	Hb24	26	M	B	11/2014	x			2 4 2 3 2 3 2 3 3 2 5 3 3 6 3 4 2 4 3 5 >20 14 5 3		Non-Beijing
40												
141	–	Eb12	76	F	B	01/2011			x	4 4 2 3 3 3 4 6 4 4 7 5 3 8 3 3 3 4 7 7 8 14 14 10	x	Modern Beijing
142	–	Gd02	23	F	D	01/2013			x	4 4 2 3 3 3 4 6 4 4 7 4 3 8 3 3 3 4 7 7 8 14 14 10	x	Modern Beijing
41												
143	–	Db04	76	M	B	04/2010			x	4 4 2 3 3 3 4 8 4 4 7 5 3 8 3 3 3 4 7 8 8 14 14 9	x	Modern Beijing
144	–	Ga41	25	M	A	11/2013			x	4 4 2 3 3 3 4 9 4 4 7 5 3 8 3 3 3 4 7 8 8 14 14 9	x	Modern Beijing
42												
145	34	Gc07	91	F	C	06/2013	x			4 4 2 3 3 3 4 7 4 4 9 5 3 9 3 3 3 4 7 6 1 12 16 9		Modern Beijing
146	34	Gc09	61	F	C	08/2013	x			4 4 2 3 3 3 4 7 4 4 9 5 3 9 3 3 3 4 7 6 1 12 16 9		Modern Beijing
147	34	Hc02	24	F	C	01/2014	x			4 4 2 3 3 3 4 7 4 4 9 5 3 9 3 3 3 4 7 6 1 12 16 9		Modern Beijing
148	34	Hc03	55	F	C	01/2014	x			4 4 2 3 3 3 4 7 4 4 9 5 3 9 3 3 3 4 7 6 1 12 16 9		Modern Beijing
149	34	Hc22	30	F	C	04/2014	x			4 4 2 3 3 3 4 7 4 4 9 5 3 9 3 3 3 4 7 6 1 12 16 9		Modern Beijing
150	34	Ic03	51	F	C	03/2015	x			4 4 2 3 3 3 4 7 4 4 9 5 3 9 3 3 3 4 7 6 1 12 16 9		Modern Beijing
151	34	Ic05	54	M	C	04/2015	x			4 4 2 3 3 3 4 7 4 4 9 5 3 9 3 3 3 4 7 6 1 12 16 9		Modern Beijing
43												
152	35	Fa05	71	F	A	02/2012		x		4 4 2 3 1 3 3 5 4 4 7 5 3 8 5 2 4 4 7 9 9 18 12 11		ST3
153	35	Ha01	73	F	A	01/2014		x		4 4 2 3 1 3 3 5 4 4 7 5 3 8 5 2 4 4 7 9 9 18 12 11		ST3

Preliminary cluster, strain no.†	Cluster	Isolate	Age, y	Sex	Residential area	Registration month/year	Epidemiologic link			VNTR profile of 24$_{Beijing}$-VNTR‡	SLV	*M. tuberculosis* lineage
							Linked	Possibly linked	Not linked			
44												
154	36	Fb10	70	M	B	11/2012	x			4 4 2 3 3 3 4 >20 3 4 7 5 4 8 3 3 3 4 7 8 5 14 14 12		Modern Beijing
155	36	Hb06	28	F	B	03/2014		x		4 4 2 3 3 3 4 >20 3 4 7 5 4 8 3 3 3 4 7 8 5 14 14 12		Modern Beijing
156	36	Hb15	79	M	B	07/2014	x			4 4 2 3 3 3 4 >20 3 4 7 5 4 8 3 3 3 4 7 8 5 14 14 12		Modern Beijing
45												
157	37	Gb27	93	F	B	11/2013			x	3 4 2 2 3 3 4 5 4 4 7 5 3 9 4 3 3 2 10 9 8 12 9 8		ST11/26
158	–	Hc14	62	F	C	05/2014			x	3 4 2 2 3 3 4 5 4 4 7 5 3 9 4 2 3 2 10 9 8 12 9 8	x	ST11/26
159	37	Ia27	29	M	A	01/2015			x	3 4 2 2 3 3 4 5 4 4 7 5 3 9 4 3 3 2 10 9 8 12 9 8		ST11/26
46												
160	38	Fa18	82	F	A	06/2012	x			4 4 4 3 3 3 3 3 4 2 7 5 3 3 4 3 3 4 7 8 8 12 18 17		STK
161	38	Hb18	55	F	B	08/2014	x			4 4 4 3 3 3 3 3 4 2 7 5 3 3 4 3 3 4 7 8 8 12 18 17		STK
47												
162	39	Ga31	83	M	A	09/2013	x			1 4 2 1 3 3 1 4 3 2 4 3 8 5 3 3 2 4 11 4 2 5 3 2		Non-Beijing
163	39	Ha19	82	F	A	07/2014	x			1 4 2 1 3 3 1 4 3 2 4 3 8 5 3 3 2 4 11 4 2 5 3 2		Non-Beijing
48												
164	–	Hc09	83	M	C	02/2014			x	3 4 2 3 3 4 3 7 4 4 7 5 3 2 5 4 3 5 4 10 8 12 12 13	x	ST25/19
165	–	Ha33	81	M	A	04/2014			x	3 4 2 3 3 4 3 7 4 4 7 5 3 2 5 4 3 5 4 10 8 12 12 11	x	ST25/19
49												
166	40	Ca36	85	F	A	12/2009	x			6 4 2 3 1 3 3 7 4 4 8 5 3 7 5 2 5 4 7 10 >20 16 18 13		ST3
167	40	Ia06	92	M	A	03/2015	x			6 4 2 3 1 3 3 7 4 4 8 5 3 7 5 2 5 4 7 10 >20 16 18 13		ST3
50												
168	41	Hb19	89	M	B	09/2014			x	4 4 2 3 3 3 3 3 4 2 6 3 4 7 4 3 3 4 7 8 8 13 18 13		STK
169	41	Ia22	88	M	A	09/2015			x	4 4 2 3 3 3 3 3 4 2 6 3 4 7 4 3 3 4 7 8 8 13 18 13		STK
51												
170	–	Fb01	74	M	B	01/2012			x	4 4 2 3 3 3 3 3 4 2 7 3 4 7 4 3 3 4 7 8 8 13 16 11	x	STK
171	–	Ia23	94	M	A	11/2015			x	4 4 2 3 3 3 3 3 4 2 7 3 4 7 4 3 3 4 7 8 8 13 16 12	x	STK
52												
172	42	Hc26	87	F	C	11/2014			x	3 4 1 2 5 1 2 2 3 4 1 3 3 8 4 1 3 2 13 7 >20 5 9 3		Non-Beijing
173	42	Ia26	87	M	A	12/2015			x	3 4 1 2 5 1 2 2 3 4 1 3 3 8 4 1 3 2 13 7 >20 5 9 3		Non-Beijing

*SLV, single-locus variant; –, the case was not included in a cluster. Underlining indicates the position of the SLV profile.

† Because we defined that a strain can belong to a preliminary cluster, there were cases in which strains of ≥2 loci difference were included in the same preliminary cluster.

‡The 24 digits indicate the VNTR profile according to the following aliases (loci) set order: Mtub04 (424), ETR C (577), MIRU4 (580), MIRU40 (802), MIRU10 (960), MIRU16 (1644), Mtub21 (1955), QUB-11b (2163b), ETR A (2165), Mtub30 (2401), MIRU26 (2996), MIRU31 (3192), Mtub39 (3690), QUB-26 (4052), QUB-4156 (4156), Mtub24 (2074), V2372 (2372), QUB-15 (3155), QUB-3336 (3336), QUB-18 (1982), QUB-11a (2163a), QUB-3232 (3232), V3820 (3820), and V4120 (4120). Additionally, as for the copy number represented by ">20," we confirmed the concordance of PCR product size within the cluster by using agarose electrophoresis.

Technical Appendix 1 **Table 2.** Characteristics of three large tuberculosis clusters in Yamagata Prefecture, Japan, 2009–2015

Characteristics of the source case-patient	Cluster 12, N = 35	Cluster 26, N = 14	Cluster 34, N = 8
Isolate number	Aa08	Fb05	Not acquired
Age, y	20s	20s	90s
Sex	Male	Male	Female
Site of disease	Pulmonary (sputum smear positive)	Pulmonary (sputum smear positive)	Lung cancer (TB was not diagnosed)*
Notification date (death date)	November 2007	November 2012	(September 2012)
Time between symptom onset and date of diagnosis (time between symptom onset and date of death)	35 months†	3 months	(21 months)†
Frequently visited places	Two workplaces (changed job once during onset), club team and its events, pachinko parlors‡	A welfare daycare center, pachinko parlors‡	A health clinic (Hospital 1 within cluster 34 in Technical Appendix 1 Figure 2), A hospital admitted (Hospital 2 within cluster 34 in Technical Appendix 1 Figure 2)
Remarks	The source case-patient had a medical checkup annually after 2003, but his TB was not diagnosed properly.	The source case-patient was mildly mentally handicapped.	The probable source case-patient was suspected of having lung cancer by tumor marker diagnosis in July 2010. Steroid therapy for terminal care was performed at Hospital 2 for 5 days before she died. Suctioning of sputum was applied frequently for the patient without adequate standard precautions of healthcare workers.

*After $24_{Beijing}$-VNTR typing, a public health center confirmed the case-patient's typical tuberculous cavity in a chest radiograph taken in late August 2012, slightly before the patient's death (data not shown). VNTR, variable-number tandem-repeat.

†Public health centers estimated the duration of symptoms by retrospective viewing of chest radiographs (data not shown).

‡Pachinko parlors, crowded gambling halls in Japan, are well attended by persons ≥18 years of age.

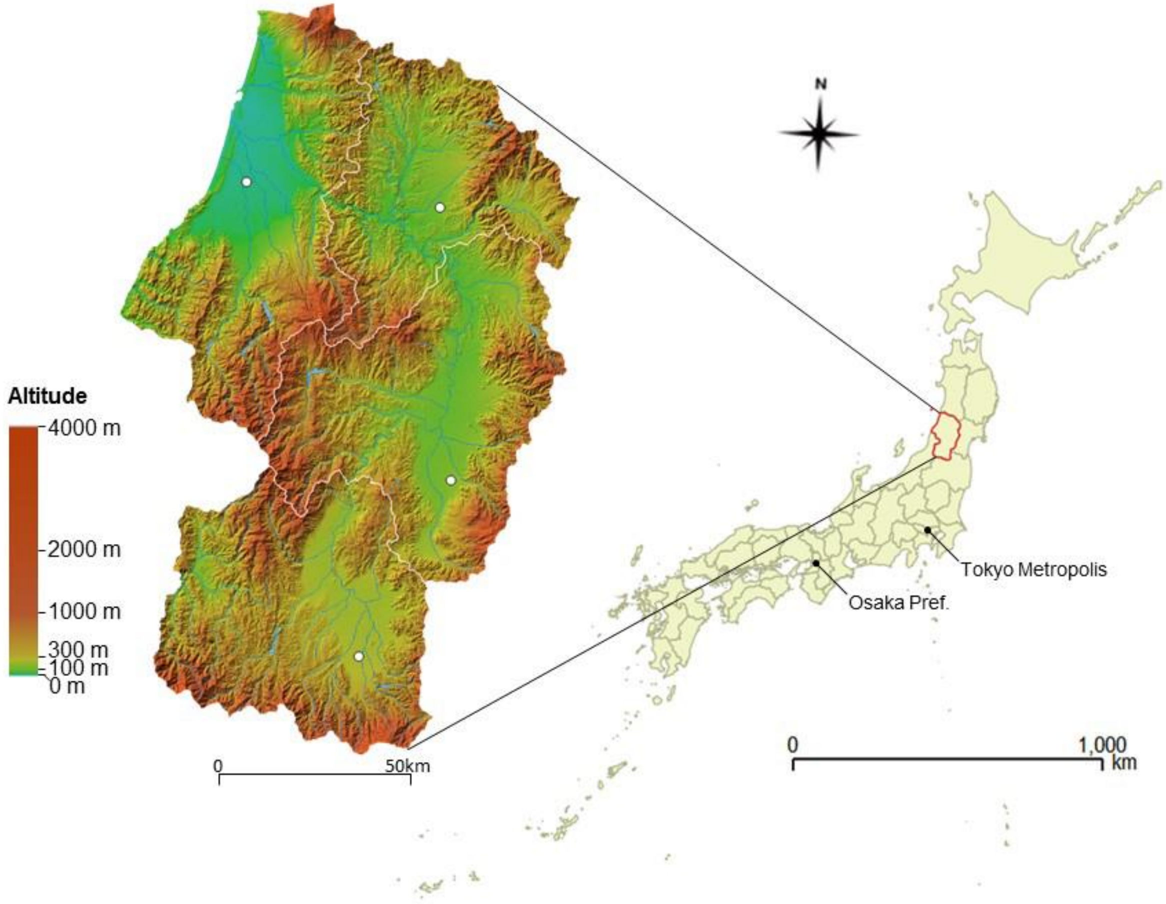

Technical Appendix 1 **Figure 1.** Topographic map of Yamagata Prefecture, Japan. About 85% of the landforms are mountains. Boundaries among the four areas (white line) are separated mainly by the mountains. White circles show the locations of Public Health Centers in respective areas. Map source: The Geospatial Information Authority of Japan, Ministry of Land, Infrastructure, Transport and Tourism (http://maps.gsi.go.jp/development/ichiran.html)

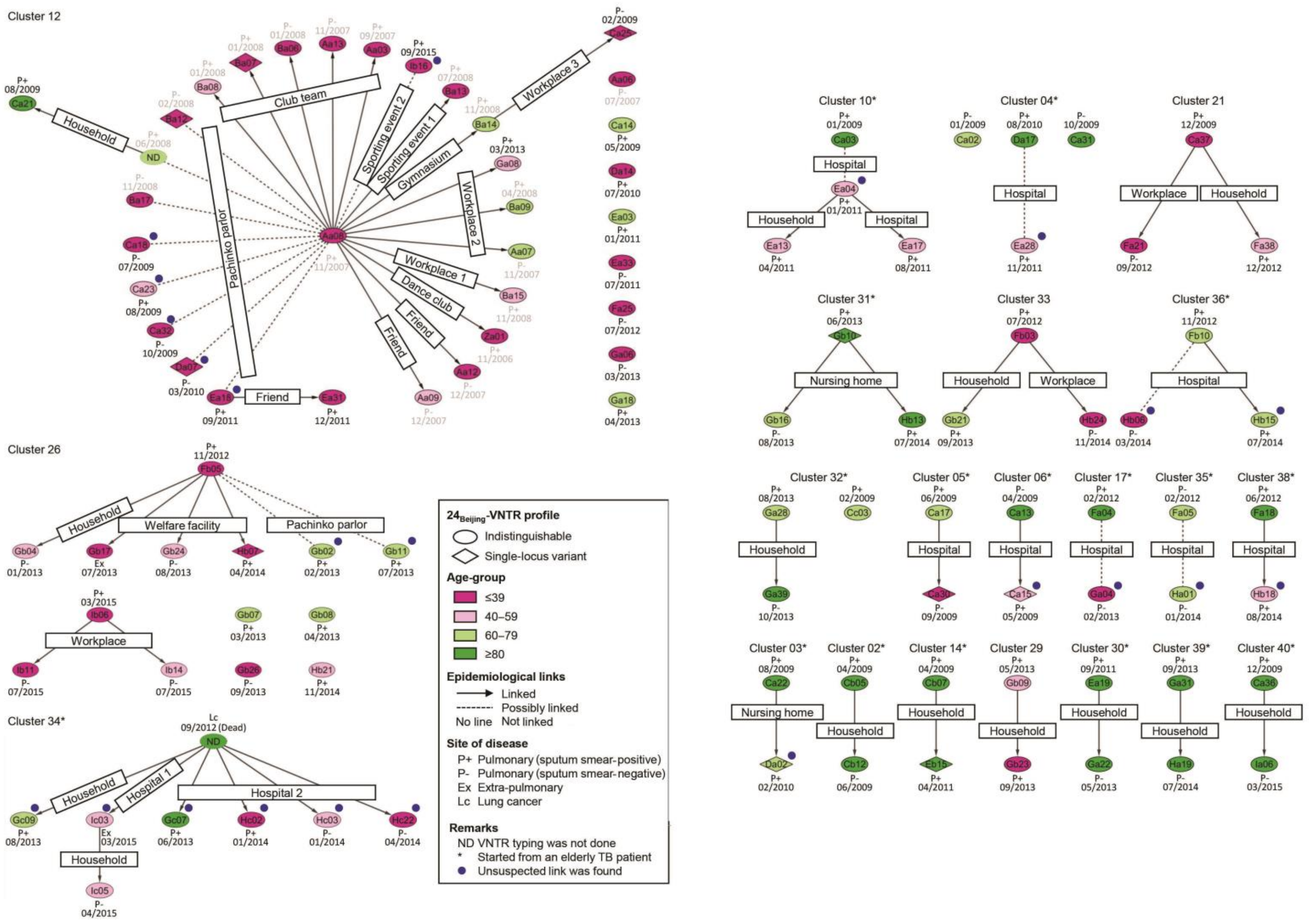

Technical Appendix 1 **Figure 2.** Twenty-two clusters with epidemiologic links between patients, Yamagata Prefecture, Japan, 2009–2015. In 86 cases depicted, 70 cases are graded as linked or possibly linked; 15 cases are nonlinked; a case (the probable source case of cluster 34) is

excluded from the study because variable-number tandem-repeat (VNTR) typing of the case could not be done. Cluster 12 contains 18 cases that are outside of the study period (shown by gray text). Ovals and diamonds denote individual cases in each cluster; numbers inside symbols are patient identification codes. Profiles of $24_{Beijing}$-VNTR typing, patient age groups, epidemiologic links in each cluster, and patient disease sites are shown in the key. Transmission settings for linked cases are shown within rectangles. Patient disease sites and case notification dates are shown above/below the case symbol. Blue dots signify confirmation of the epidemiologic link by in-depth contact tracings after $24_{Beijing}$-VNTR typing. Asterisks indicate clusters that began with a tuberculosis source patient who was ≥60 years of age.
