# Supplementary material for: Mycobacterium tuberculosis Transmission among Elderly Persons, Yamagata Prefecture, Japan, 2009–2015
Source: Emerg Infect Dis. 2017 Mar;23(3):448–55. doi: 10.3201/eid2303.161571 (PMC5382749; doi:10.3201/eid2303.161571)
Supplement: Technical Appendix 2 — Self-administered questionnaires used to elicit responses related to residential and travel history, transportation, and places of social aggregation from persons with culture-confirmed tuberculosis, Yamagata Prefecture, Japan, 2009–2015. [file 16-1571-Techapp-s2.pdf]

Article DOI: http://dx.doi.org/10.3201/eid2303.161571

Mycobacterium tuberculosis Transmission among Elderly Persons, Yamagata Prefecture, Japan, 2009–2015

Technical Appendix 2

Public health centers in Yamagata Prefecture, Japan, use 2 versions of a questionnaire (1 long and 1 short) to gather data from tuberculosis case-patients. The long version contains multiple-choice and open-ended questions. The short version contains only the multiple-choice questions from the long questionnaire. Public health centers chose which questionnaire to use, depending on the patient's willingness and ability to respond.

Long Questionnaire

Questionnaire for early findings of tuberculosis

This questionnaire is intended to show us the places where tuberculosis transmissions occur. Please respond with information about your past lifestyle. We would like to use your cooperation to decrease tuberculosis infection in Yamagata.

Please feel free to contact us if you have any questions.

Public health center:__________ Tel.:__________________ Person in charge:_______________

I. General questions

Q1. Did you know about "tuberculosis" before your hospital stay (or visit)?
Please check the box (☐).

☐ Yes ☐ No

Q2. Do you remember any tuberculosis patients around you in the recent past?

☐ Yes ☐ No

➔ If "Yes," please explain a little about that patient(s).

(e.g., my grandfather developed tuberculosis in 2015.)

Q3. Before you were diagnosed with tuberculosis, had you experienced any symptom such as cough, retention of sputum, or fever?

☐ Yes ☐ No

➔ If "Yes," please write the period that you started to have those symptoms.

➔ If "No," please write the period that you were diagnosed with tuberculosis.

Year: __________, Month: _________

II. Questions about your lifestyle of the last two years

Most patients show onset of tuberculosis (or have it discovered) within two years of their infection. In this section, please answer about your lifestyle for the last two years.

Q4. Please check all the facilities that you have visited at least once within the last two years.

- ☐ Convenience store
- ☐ Pachinko parlor
- ☐ Parlors of Japanese chess, mah-jong, or game of go
- ☐ Theater
- ☐ Public bathhouse
- ☐ Drinking spot (e.g., bar, pub)
- ☐ Karaoke bar
- ☐ Culture lesson (e.g., cooking class, karaoke class)
- ☐ Barber shop
- ☐ Religious space (e.g., church, temple)

Continued on next page

- ☐ Internet cafe
- ☐ Indoor sports facility
- ☐ Welfare facility (e.g., nursing home, facilities for the disabled)
- ☐ Ceremonial function (e.g., bridal, burial)
- ☐ Homes of friends, or close relatives
- ☐ Amusement arcade
- ☐ Academy, cram school
- ☐ Neighborhood meeting (e.g., neighborhood association, volunteer fire company)
- ☐ Dormitory for student or office worker
- ☐ Other place ( )

➔ If you checked more than one place, proceed to Q5.

➔ If you visited none of the places above, proceed to Q6.

Q5. Do you remember meeting someone who had a bad cough or a sudden decrease in weight?

☐ Yes ☐ No

➔ If "Yes," please write when and where you met them.

( )

Q6. Please answer the top four places where you have stayed for a long period within the last two years. Select the number of places from "1. Home," "2. Company," "3. School," "4. Amusement facility," and "5. Other place." Please answer separately for weekdays and holidays. In this question, "4. Amusement facility" means a place where you feel amused or relaxed (e.g., drinking spot, karaoke bar, and pachinko parlor).

	Weekday	Holiday
1st		
2nd		
3rd		
4th		

Q7. Within "4. Amusement facility" and "5. Other place" of Q6, please write the top three specific facilities that you have often visited. Please answer separately, divided into weekdays and holidays.

Weekday

	Name of facility	Frequency of visit	Sojourn time per visit, and visited time zone
1st			
2nd			
3rd			

Holiday

	Name of facility	Frequency of visit	Sojourn time per visit, and visited time zone
1st			
2nd			
3rd			

Q8. Please check all the transportation that you have used at least once within the last two years.

☐ Family car ☐ Bus ☐ Train
☐ Taxi ☐ Chauffeur service ☐ Plane
☐ Company car ☐ Car of welfare service ☐ Others ( )

Q9. Please check all the clinical departments where you have visited (including visit the office, attendance, and see someone) at least once within the last two years.

☐ Internal medicine ☐ Surgery ☐ Dermatology
☐ Dentistry ☐ Ophthalmology ☐ Urinology
☐ Otorhinolaryngology ☐ Orthopedics ☐ Plastic surgery
☐ Psychiatry ☐ Radiology ☐ Psychosomatic medicine
☐ Obstetrics and Gynecology ☐ Others ( )

Q10. Did you change your residence within the last two years?

☐ Yes ☐ No

Q11. Have you stayed more than one month at a facility other than your home (e.g., nursing home, training institute) within the last two years?

☐ Yes ☐ No

➔ If "Yes," please write the place(s) and the period(s) that you have stayed.

( )

Q12. Please check all the regions you have visited at least once within the last two years.

Areas of Yamagata other than the residence area: ☐ Murayama, ☐ Mogami, ☐ Oitama, ☐ Shonai

☐ Miyagi Pref. ☐ Fukushima Pref. ☐ Akita Pref.
☐ Aomori Pref. ☐ Iwate Pref. ☐ Hokkaido
☐ Kanto region ☐ Chubu region ☐ Kinki region
☐ Chugoku region ☐ Shikoku region ☐ Kyushu region

➔ If you checked more than one region, proceed to Q13.

➔ If you have not visited any region above, proceed to Q14.

Q13. Within the regions you checked at Q12, please provide details (when and how long did you visit, etc.) of the top three places that you can recall.

	Place	Details of visit
1st		
2nd		
3rd		

III. Questions about your lifestyle since you were born

Some people show onset of tuberculosis after a lapse of more than two years (sometimes after many decades) following an earlier tuberculosis infection. For tuberculosis control, it is very important for us to consider the places of tuberculosis infection. In this section, please answer about your lifestyle since you were born.

Q14. Where did you live after you were born? Please mark the undernoted figure after reading the following notes.

- Separate the figure by a vertical line, and write the name of your city (e.g., Tendo, Yamagata).
- If you have stayed at foreign countries, write the name of the province (e.g., Yunnan, China).
- If you have stayed at a facility such as a nursing home, write the facility address.

Current age _______ years old

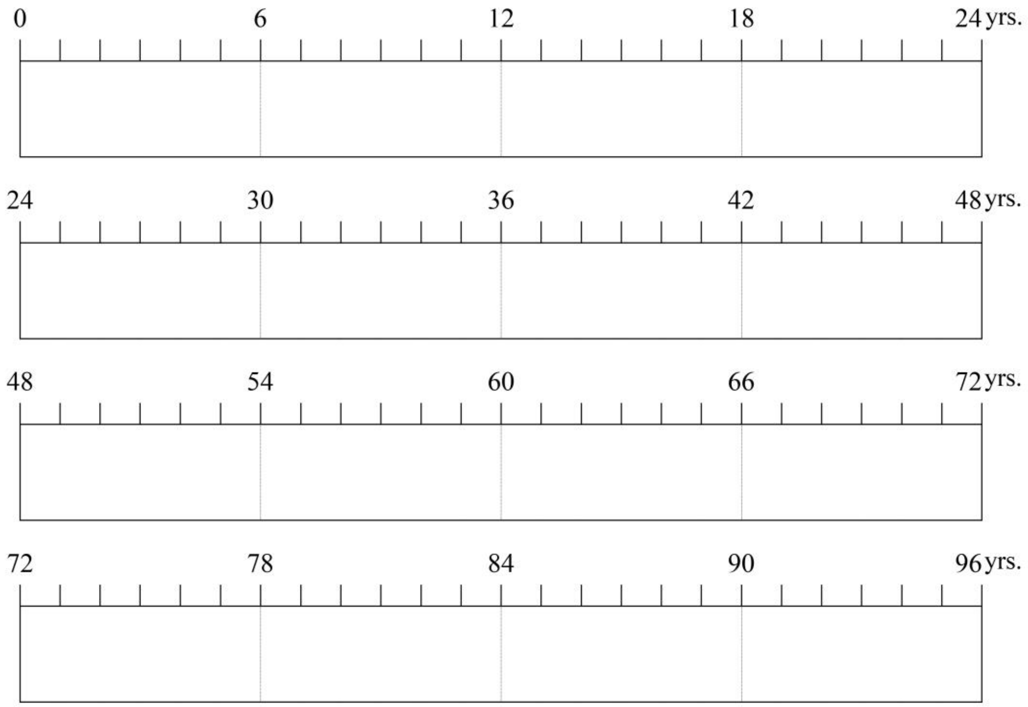

Q15. Please check all the workplaces at which you have worked.

☐ Medical agency	☐ Welfare facility	☐ Restaurant, drinking spot
☐ Pachinko parlor	☐ School	☐ Cram school
☐ Construction industry	☐ Delivery business	☐ Hotel business

Q16. Have you ever gone abroad?

☐ Yes ☐ No

➔ If "Yes," please provide some details about when and where you went abroad.

( )

Q17. Please write the schools and workplaces that you attended (or worked). Please turn off an undernoted figure separating the figure by a vertical line. Write the specific names of schools and workplaces (e.g., X kindergarten, Y corporation).

0 6 12 18 24 yrs.

24 30 36 42 48 yrs.

48 54 60 66 72 yrs.

The questionnaire is finished. Thank you for your cooperation. We would appreciate your further cooperation with any additional questions.

WE WILL END TB

WHO, 2016

Short Questionnaire

Questionnaire for early findings of tuberculosis

This questionnaire is intended to show us the places where tuberculosis transmissions occur. Please respond with information about your past lifestyle. We would like to use your cooperation to decrease tuberculosis infection in Yamagata.

Please feel free to contact us if you have any questions.

Public health center:__________ Tel.:__________________ Person in charge:_______________

I. Questions about your lifestyle of the last two years

Most patients show onset of tuberculosis (or have it discovered) within two years of their infection. In this section, please answer about your lifestyle for the last two years.

Q1. Please check the box (□) to all the facilities that you have visited at least once within the last two years.

- □ Convenience store
- □ Pachinko parlor
- □ Parlors of Japanese chess, mah-jong, or game of go
- □ Theater
- □ Public bathhouse
- □ Internet cafe
- □ Indoor sports facility
- □ Welfare facility (e.g., nursing home, facilities for the disabled)
- □ Ceremonial function (e.g., bridal, burial)
- □ Homes of friends, or close relatives
- □ Drinking spot (e.g., bar, pub)
- □ Karaoke bar
- □ Culture lesson (e.g., cooking class, karaoke class)
- □ Barber shop
- □ Religious space (e.g., church, temple)
- □ Amusement arcade
- □ Academy, cram school
- □ Neighborhood meeting (e.g., neighborhood association, volunteer fire company)
- □ Dormitory for student or office worker
- □ Other place (   )

Q2. Did you change your residence within the last two years?

□ Yes □ No

Q3. Have you stayed more than one month at a facility other than your home (e.g., nursing home, training institute) within the last two years?

□ Yes □ No

Q4. Please check all the transportation that you have used at least once within the last two years.

- □ Family car
- □ Taxi
- □ Company car
- □ Bus
- □ Chauffeur service
- □ Car of welfare service
- □ Train
- □ Plane
- □ Others (   )

Q5. Please check all the clinical departments where you have visited (including visit the office, attendance, and see someone) at least once within the last two years.

☐ Internal medicine ☐ Surgery ☐ Dermatology

☐ Dentistry ☐ Ophthalmology ☐ Urinology

☐ Otorhinolaryngology ☐ Orthopedics ☐ Plastic surgery

☐ Psychiatry ☐ Radiology ☐ Psychosomatic medicine

☐ Obstetrics and Gynecology ☐ Others (   )

II. Questions about your lifestyle since you were born

Some people show onset of tuberculosis after a lapse of more than two years (sometimes after many decades) following an earlier tuberculosis infection. For tuberculosis control, it is very important for us to consider the places of tuberculosis infection. In this section, please answer about your lifestyle since you were born.

Q6. Please check all the workplaces at which you have worked.

☐ Medical agency ☐ Welfare facility ☐ Restaurant, drinking spot

☐ Pachinko parlor ☐ School ☐ Cram school

☐ Construction industry ☐ Delivery business ☐ Hotel business

Q7. Have you ever gone abroad?

☐ Yes ☐ No

Q8. Do you remember any tuberculosis patients around you in the recent past?
(e.g., my grandfather developed tuberculosis)

☐ Yes ☐ No

The questionnaire is finished. Thank you for your cooperation. We would appreciate your further cooperation with any additional questions.

WE WILL END TB

WHO, 2016
